# Baseline Ferritin and Body Composition as Correlates of Longitudinal Metabolic Changes During an Energy-Restricted Mediterranean-Style Intervention Across Three Overweight Phlogistic-Related Clinical Conditions

**DOI:** 10.3390/nu18183044

**Published:** 2026-09-17

**Authors:** Begoña de Cuevillas, Amanda Cuevas-Sierra, Andrea Higuera-Gómez, Victor Moreno-Torres, Susana Mellor-Pita, María Martínez-Urbistondo, Raquel Castejón, Daniel De Luis-Román, J. Alfredo Martínez

**Affiliations:** 1 Precision Nutrition and Cardiometabolic Health, IMDEA-Food Institute (Madrid Institute for Advanced Studies), Campus of International Excellence (CEI) UAM+CSIC, 28049 Madrid, Spain; begona.decuevillas@nutricion.imdea.org (B.d.C.); andrea.higuera@nutricion.imdea.org (A.H.-G.); 2 Department of Endocrinology, Clínica Universidad de Navarra, 28027 Madrid, Spain; 3 Department of Pharmacy and Nutrition, Faculty of Biomedical and Health Sciences, Universidad Europea de Madrid, Campus de Villaviciosa de Odón, 28670, Madrid, Spain; 4 Internal Medicine Service of Puerta de Hierro Majadahonda University Hospital, 28222 Madrid, Spain; victor.moreno.torres.1988@gmail.com (V.M.-T.); susanamellor@hotmail.com (S.M.-P.); mmurbistondo@gmail.com (M.M.-U.); raquel.castejon@salud.madrid.org (R.C.); 5 Health Sciences School and Medical Centre, International University of the Rioja (UNIR), 28224 Madrid, Spain; 6 Centre of Endocrinology and Nutrition, University of Valladolid, 47003 Valladolid, Spain; dluisro@saludcastillayleon.es; 7 Department of Endocrinology and Nutrition, University Clinical Hospital, University of Valladolid, 47002 Valladolid, Spain; 8 Centro de Investigación Biomédica en Red de la Fisiopatología de la Obesidad y Nutrición (CIBERobn), Instituto de Salud Carlos III, 28029 Madrid, Spain

**Keywords:** ferritin, body composition, overweight and obesity, long COVID, metabolic syndrome, Mediterranean diet, prospective cohort

## Abstract

Background/Objectives: Chronic inflammation accompanies obesity, but correlations of longitudinal changes during dietary follow-up remain unclear. We described inflammatory profiles and examined associations of baseline ferritin and body composition with changes during a 32-week energy-restricted Mediterranean-style intervention. Methods: This prospective cohort included 190 adults classified as healthy overweight (*n* = 49), long COVID with overweight (*n* = 108), or metabolic syndrome (MS) with overweight (*n* = 33); 61 healthy normal-weight participants without long COVID or metabolic syndrome contributed to baseline comparisons only. The program prescribed a 30% energy deficit using a Mediterranean-style food-exchange system. Anthropometric, biochemical, inflammatory, lifestyle, and hepatic markers were assessed. MANOVA and multivariable regression examined inflammatory profiles and ferritin-change correlates. Results: Participants with overweight/obesity had a less favorable metabolic and inflammatory profile than normal-weight participants. Baseline ferritin was 100.5 (53.5–178.5), 76.0 (34.0–137.0), and 149.5 (102.0–245.0) ng/mL in the healthy overweight, long-COVID, and MS groups, respectively (omnibus *p* < 0.001). Mean changes were 8.0 ± 36.4, 3.3 ± 32.6, and −22.0 ± 58.8 ng/mL, respectively (omnibus *p* = 0.039), with the MS group showing greater decreases. Sex and muscle mass were associated with the multivariate inflammatory profile (both *p* < 0.001). Clinical group, baseline ferritin, muscle-mass change, MEDAS change, and Fatty Liver Index change were associated with ferritin change, although explanatory performance was limited (adjusted R^2^ = 0.16). Conclusions: Baseline ferritin and body composition were correlates of heterogeneous inflammatory-metabolic profiles and ferritin changes. These hypothesis-generating associations do not establish causal or mechanistic effects and require controlled confirmation with comprehensive iron-status assessment.

## 1. Introduction

Chronic low-grade inflammation is a persistent, dysregulated immune response that contributes to cardiometabolic disease, diabetes, cardiovascular disorders, and impaired health-related quality of life [1,2,3]. Unlike acute inflammation, which is protective and self-limited, chronic activation of inflammatory pathways can promote tissue dysfunction and metabolic deterioration. Aging is associated with persistent immune activation [4,5], while biological sex also shapes inflammatory responses and adipose-tissue function [6]. In obesity, adipose-tissue expansion and immune-cell infiltration increase the production of pro-inflammatory mediators, including interleukin-6 (IL-6) and tumor necrosis factor-alpha (TNF-alpha) [7,8]. Physical activity has been associated with lower systemic inflammation [9], whereas lower skeletal muscle mass and function have been associated with higher CRP, IL-6, and TNF-alpha concentrations [10]. Obesity-related inflammation and insulin resistance also intersect with hepatic steatosis [11].

Inflammatory phenotypes may differ across obesity-related clinical conditions. Metabolic syndrome (MS) combines central adiposity, insulin resistance, dyslipidemia, hypertension, and other disturbances associated with low-grade immune activation [12]. Long COVID is characterized in a subset of patients by persistent immune dysregulation, endothelial and coagulation abnormalities, metabolic perturbations, and prolonged symptoms after SARS-CoV-2 infection [13,14]. Ferritin may be relevant in both settings because it reflects iron stores but also behaves as an acute-phase reactant [15]. Hyperferritinemia has been associated with insulin resistance, hepatic fat accumulation, and MS [16,17], while altered ferritin concentrations have also been described during and after COVID-19 [13,14]. Comparing healthy overweight, long-COVID overweight, and MS overweight groups may therefore help identify whether distinct inflammatory-metabolic contexts are associated with different baseline profiles and longitudinal responses.

Dietary exposures have been associated with variation in inflammatory signaling, oxidative stress, and gut-microbiota composition [18,19]. Mediterranean-style dietary patterns, rich in vegetables, fruit, legumes, nuts, fish, olive oil, fiber, polyphenols, and antioxidant vitamins, have been associated with lower inflammatory biomarker concentrations and improved cardiometabolic risk [20,21,22,23,24]. Conversely, Western dietary patterns are characterized by high intakes of saturated fat, refined sugars, and ultra-processed foods [25]. Evidence from randomized trials and meta-analyses indicates that caloric restriction can reduce adiposity, including visceral fat, and may modestly lower selected inflammatory biomarkers in adults with overweight or obesity [16,26,27]. These findings support consideration of both dietary quality and energy restriction when describing body-composition and inflammatory-marker variation.

Baseline inflammatory and metabolic status may be associated with variation in longitudinal responses through several non-exclusive pathways. Individuals with greater visceral adiposity, hepatic dysfunction, insulin resistance, or altered iron handling may begin with higher biomarker concentrations and show different patterns of change. Ferritin is informative but non-specific: variation may reflect inflammation, iron stores, dietary heme iron, liver status, sex, reproductive status, medication, or other clinical factors. Consensus criteria for metabolic hyperferritinemia emphasize ferritin elevation in the context of metabolic dysfunction after assessment of alternative causes and, where available, evidence of iron accumulation or related organ damage [17]. Accordingly, ferritin was evaluated here as a correlate rather than as a direct measure of inflammation, iron overload, or a causal dietary effect. Although ferritin has been associated with visceral adiposity, insulin resistance, hepatic dysfunction, and sex-related metabolic differences, its relationship with body-composition changes during dietary follow-up remains incompletely characterized, particularly across distinct overweight-related clinical phenotypes.

Despite increasing evidence linking diet, inflammation, and metabolic health, the combined evaluation of ferritin and body composition as correlates of longitudinal response remains limited. In particular, few studies have jointly examined baseline ferritin, body composition, and longitudinal metabolic responses across overweight populations with long COVID, MS, and no diagnosed inflammatory comorbidity. The present exploratory study aimed to compare baseline inflammatory profiles among these groups and to evaluate metabolic and inflammatory changes during an approximately 32-week energy-restricted Mediterranean-style intervention. We hypothesized that participants with a more adverse baseline inflammatory-metabolic phenotype, particularly higher ferritin and visceral adiposity, would show a different pattern of longitudinal response, and that ferritin changes would be associated with baseline ferritin, body-composition changes, dietary adherence, and hepatic-metabolic status.

## 2. Materials and Methods

### 2.1. Experimental Approach

This study was conducted within the METAINFLAMMATION-CM project (ref. Y2020/BIO-6600), a prospective longitudinal cohort study designed to examine chronic inflammation in relation to excess body weight. Baseline assessments included clinical evaluation, anthropometry and body composition, fasting biochemical and inflammatory measurements, and lifestyle and health-related questionnaires. Participants with overweight/obesity who entered the nutritional program received an individualized energy-restricted Mediterranean-style diet for approximately 32 weeks. A face-to-face follow-up was scheduled at week 8, and monthly telephone contacts were conducted from diet initiation to assess adherence, identify possible complications, answer questions, and reinforce the dietary plan. Final in-person anthropometric, biochemical, inflammatory, and questionnaire assessments were performed at the end of the intervention. The normal-weight control group underwent baseline assessment only and did not receive the intervention. The health-related questionnaires were administered at baseline and at the final in-person evaluation; dietary adherence was additionally reviewed during the scheduled follow-up contacts.

### 2.2. Participants

**Figure 1:**
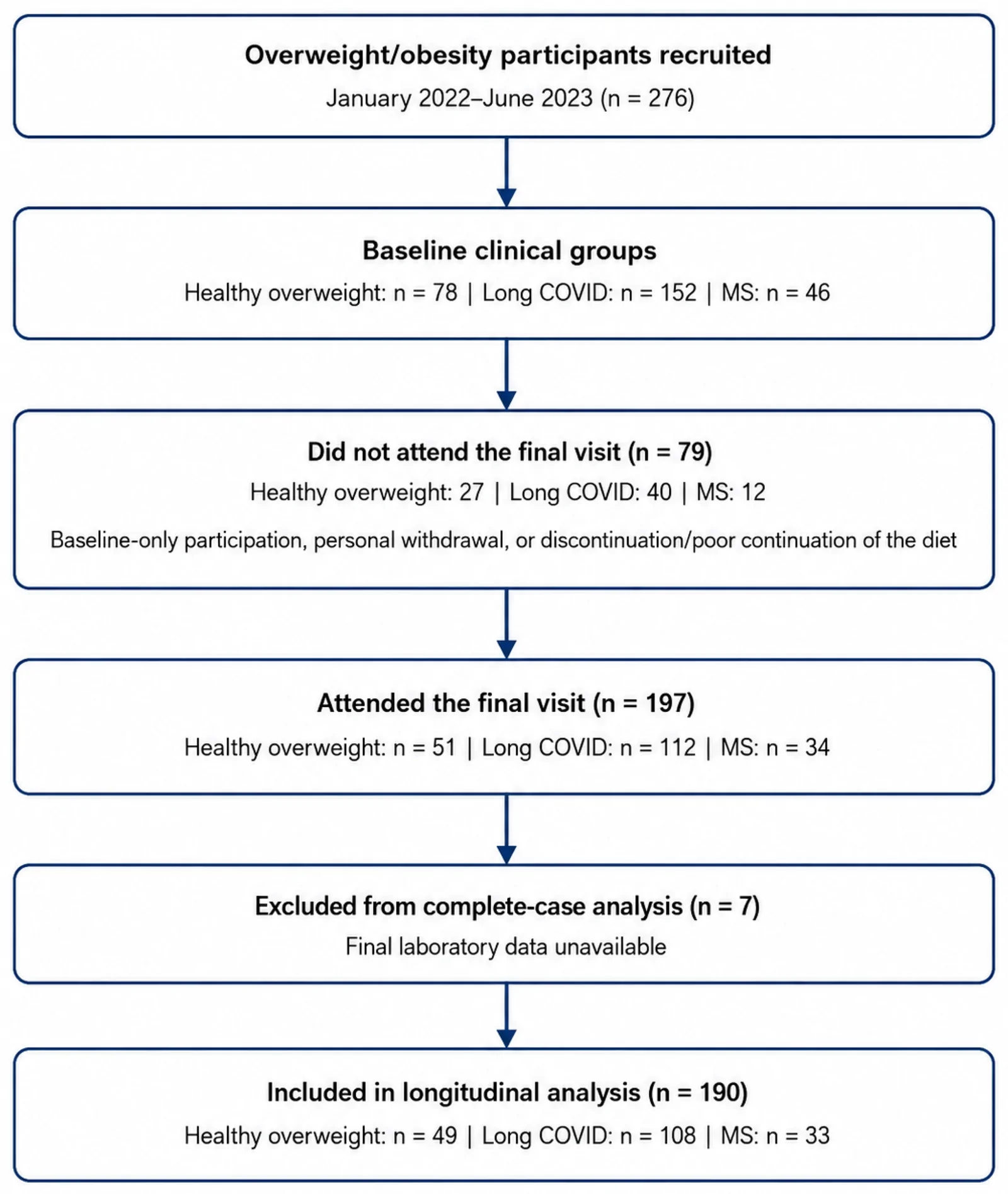
Flow of participants with overweight/obesity from recruitment to the longitudinal complete-case analysis. MS, metabolic syndrome.

Adult men and women of Caucasian or Hispanic origin were consecutively recruited from the Internal Medicine Service of Puerta de Hierro Majadahonda University Hospital (Madrid, Spain) between January 2022 and June 2023. A total of 276 participants with overweight/obesity were initially recruited: 78 healthy overweight, 152 long-COVID overweight, and 46 MS participants. Of these, 197 attended the final visit (51, 112, and 34, respectively). Seven attendees lacked the final laboratory data required for the present analyses, leaving 190 participants in the longitudinal complete-case sample (49, 108, and 33, respectively). A further 61 normal-weight participants were available for baseline comparisons. Reasons for not completing the final visit included choosing baseline-only participation, personal withdrawal, and discontinuation or poor continuation of the dietary program. Participant flow is shown in Figure 1. The normal-weight comparison group comprised 61 adults with BMI 18.5–24.9 kg/m^2^ who had no diagnosis of long COVID or MS; they underwent baseline assessment only.

No outcome-specific sample size calculation was performed for the present secondary analysis. The analytical sample was derived from the broader METAINFLAMMATION-CM cohort, whose design, recruitment procedures, and clinical-group structure have been described elsewhere [12]. The broader cohort included additional clinical groups not considered in the present study, such as participants with autoimmune conditions. This analysis was restricted to the clinical groups relevant to its objectives and included all eligible participants with the data required for the corresponding baseline or longitudinal analyses. The findings should therefore be considered exploratory.

Long COVID was defined according to the World Health Organization clinical case definition and NICE guidance [28,29]. The WHO definition describes post-COVID-19 condition as symptoms occurring usually three months after the onset of COVID-19, lasting for at least two months, and not explained by an alternative diagnosis; NICE defines post-COVID-19 syndrome as symptoms continuing for more than 12 weeks after the start of acute COVID-19. Consistent with these criteria, participants in the long-COVID group were evaluated after the acute infection had resolved, were no longer SARS-CoV-2-positive, and had persistent COVID-19-related symptoms for at least 12 weeks after the onset of the acute infection. The diagnosis was confirmed by an internal medicine specialist, and the preceding acute infection had been documented by a positive polymerase chain reaction test. Thus, the long-COVID classification represented a post-acute condition rather than active SARS-CoV-2 infection. Detailed information on pharmacological treatments received during acute COVID-19 and on concomitant medication use during follow-up was not included in the present analytical dataset. MS was identified using the updated National Cholesterol Education Program Adult Treatment Panel III (NCEP ATP III) criteria, as revised by the American Heart Association/National Heart, Lung, and Blood Institute and applied in the parent METAINFLAMMATION-CM cohort [12,30,31]. Diagnosis required at least three of the following five components: waist circumference ≥102 cm in men or ≥88 cm in women; triglycerides ≥150 mg/dL or treatment for elevated triglycerides; HDL cholesterol <40 mg/dL in men or <50 mg/dL in women, or specific treatment; systolic blood pressure ≥130 mmHg, diastolic blood pressure ≥85 mmHg, or antihypertensive treatment; and fasting plasma glucose ≥100 mg/dL or treatment for elevated glucose. No single component was mandatory for diagnosis. Type 2 diabetes mellitus was considered a distinct clinical diagnosis and was not required for classification as metabolic syndrome.

Clinical diagnoses and comorbidities were obtained from the participants’ medical records and confirmed during the clinical assessment. The documented conditions considered in the present analysis included obesity, type 2 diabetes mellitus, hypertension, dyslipidemia, long COVID, and metabolic syndrome. Participants belonging to the autoimmune and other clinical groups included in the broader METAINFLAMMATION-CM cohort were not included in this secondary analysis. Additional immune-mediated conditions and individual medication regimens were not systematically analyzed. Assignment to the long-COVID or MS group reflected the principal clinical phenotype relevant to this analysis and did not imply the absence of every other comorbidity. Potential coexisting conditions were identified from medical records and clinical evaluation, but were not all available as covariates in the present secondary dataset.

Eligibility criteria were age >18 years; no prespecified upper age limit was applied; body mass index 25–51 kg/m^2^ for the overweight/obesity groups, and classification as healthy overweight, long-COVID overweight, or MS. Exclusion criteria were severe psychiatric conditions, pregnancy or breastfeeding, use of medications known to affect body weight, and unwillingness to participate. Participants’ clinical histories, biochemical measurements, and follow-up data were recorded within the institutional Electronic Health Record (EHR) system (SELENE-PdH) of Hospital Universitario Puerta de Hierro Majadahonda. The research project was institutionally identified as PI 164/21 and conducted within the METAINFLAMMATION-CM project (Y2020/BIO-6600). The protocol was approved by the hospital Research Ethics Committee (PI 164-21), complied with the Declaration of Helsinki, and all participants provided written informed consent. In addition, data derived from the participants included in this study have been deposited in the NCBI Sequence Read Archive (SRA) under BioProject accession number PRJNA1279632.

### 2.3. Nutritional Intervention

Individual energy requirements were estimated for each participant, and the prescribed diet provided an approximately 30% energy deficit relative to total energy requirements. The intervention used a Mediterranean-style food-exchange system rather than a single fixed menu. This pattern emphasized foods commonly associated in the literature with lower dietary inflammatory potential, but the term does not imply that an anti-inflammatory effect was demonstrated in the present cohort. Individual daily exchange targets were assigned across fruit, nuts, vegetables, cereals, dairy products, protein-food categories, and fats according to the prescribed energy level. Participants received food lists specifying portions per exchange and were instructed to include legumes twice weekly by replacing cereal and protein exchanges, as well as eggs and oily fish twice weekly through equivalent protein and fat substitutions. This structure was intended to promote vegetables, fruit, legumes, nuts, fish, olive oil, and minimally processed carbohydrate sources while allowing individualized food choice. Adherence and tolerability were reviewed at the week-8 visit and during monthly telephone follow-up. MEDAS was assessed as a measure of adherence to the Mediterranean dietary pattern.

### 2.4. Anthropometric Measurements

Anthropometric measurements were obtained by trained dietitians following standardized assessment protocols [32]. Body weight and body composition, including skeletal muscle mass, total body fat, and visceral fat, were determined using a bioelectrical impedance device (TANITA SC-330; Tanita Corp., Tokyo, Japan). Waist circumference (WC) was measured at the midway point between the lowest rib and the top of the iliac crest using a non-elastic tape in accordance with established clinical recommendations. Hip circumference was determined at the level of the maximum circumference of the gluteus using appropriate equipment and validated methods. Body mass index (BMI) was calculated as body weight in kilograms divided by height in meters squared. Systolic and diastolic blood pressure were assessed using an automatic calibrated sphygmomanometer according to internationally accepted guidelines.

### 2.5. Biochemical and Inflammatory Measurements

Fasting venous blood samples were obtained after 8 h of overnight fasting via venipuncture and processed at the Puerta de Hierro Majadahonda University Hospital. Routine biochemical determinations were carried out to assess glucose, total cholesterol, high-density lipoprotein cholesterol (HDL-c), triglycerides (TG), glycated hemoglobin (HbA1c), bilirubin, aspartate aminotransferase (AST), alanine aminotransferase (ALT), ferritin, transferrin, and hemoglobin using a quality-controlled automated platform (Atellica™ Solution, Siemens Healthineers, Erlangen, Germany), following the hospital’s standard operating protocols. Circulating inflammatory and metabolic markers, including C-reactive protein (CRP), fibrinogen, D-dimer, insulin, and interleukin-6 (IL-6), were quantified by enzyme-linked immunosorbent assay (ELISA) employing commercially available kits (Sigma-Aldrich, St. Louis, MO, USA) according to the manufacturers’ instructions. CRP, IL-6, fibrinogen, and D-dimer were analyzed as continuous variables; assay-specific clinical reference intervals were not used to classify participants or define inflammatory status. Insulin resistance was estimated using the Homeostatic Model Assessment of Insulin Resistance (HOMA-IR), calculated as follows: HOMA-IR = [fasting glucose (mg/dL) × fasting insulin (µU/mL)]/405. Low-density lipoprotein cholesterol (LDL-c) levels were calculated using the Friedewald formula [33]: low-density lipoprotein cholesterol = total cholesterol − high-density lipoprotein cholesterol − TG/5.

CRP and IL-6 were selected as established systemic inflammatory markers, while fibrinogen and D-dimer were included to characterize inflammation-related coagulation pathways relevant to obesity and post-viral conditions. Ferritin was examined as a clinical correlate of iron storage, acute-phase activity, and hepatic-metabolic status. Ferritin was measured only on the Atellica automated platform. Ferritin was therefore interpreted alongside CRP, IL-6, body composition, dietary variables, and liver-related indices and was not treated as an isolated measure of inflammation or iron overload.

### 2.6. Lifestyle and Health-Related Determinants

Adherence to the Mediterranean dietary pattern was assessed using a previously validated 14-item screening questionnaire for the Spanish population considering the consumption of nine food groups or nutrients [34]. Fatigue was evaluated using the FACIT-Fatigue questionnaire, a 13-item instrument previously validated for use in the Spanish population, with items addressing the severity and impact of fatigue on daily functioning [35]. Psychological distress was assessed using the Self-Reporting Questionnaire-20 (SRQ-20), a 20-item screening instrument developed by the World Health Organization (WHO) to detect symptoms of common mental disorders, which has demonstrated good reliability and validity in diverse populations and primary care settings globally [36]. Non-invasive indirect hepatic indices were computed to estimate liver steatosis and fibrosis, considering the necessary criteria and applying accepted formulas [37]. The Fatty Liver Index (FLI) was derived from a logistic function incorporating BMI, waist circumference, triglycerides, and GGT, with higher scores indicating greater likelihood of fatty liver [38]. Health-related quality of life (HRQoL) was evaluated using the validated 12-Item Short Form Health Survey (SF-12), which generates physical (PCS) and mental (MCS) component summary scores [39]. Physical activity levels were assessed using the short form of the International Physical Activity Questionnaire (IPAQ), which estimates the frequency, duration, and intensity of walking, moderate, and vigorous activities over the past seven days, expressed in metabolic equivalent minutes per week (MET-min/week) [40]. For the exploratory meat-consumption comparison, participants were categorized based on baseline MEDAS item 5, which asks whether red meat, hamburgers, or meat products are consumed less than once daily. Participants meeting this criterion were classified as lower meat consumption and those not meeting it as higher meat consumption; thus, this variable was questionnaire-based and did not represent weighed dietary records.

### 2.7. Statistical Analysis

Baseline characteristics and changes from baseline to the end of follow-up were summarized across clinical groups. Continuous-variable distributions were assessed using the Shapiro–Wilk test and graphical inspection. Data are reported as mean +/− standard deviation or median (interquartile range), as appropriate. For continuous variables, comparisons among the three overweight/obesity groups used one-way analysis of variance when parametric assumptions were satisfied and the Kruskal–Wallis test for non-normally distributed variables; comparisons between the normal-weight and combined overweight/obesity samples used the independent-samples *t*-test or Mann–Whitney U test, respectively. For the principal Kruskal–Wallis analyses of baseline ferritin and ferritin change, epsilon-squared (ε^2^) was calculated as an estimate of effect size. When the overall Kruskal–Wallis test was significant, pairwise post hoc comparisons were performed using Dunn’s test with Holm adjustment for multiple comparisons. Categorical variables were compared using the chi-squared test. For analyses involving more than two groups, omnibus *p*-values were interpreted without inferring pairwise differences when post hoc comparisons were unavailable. Change scores were summarized as mean +/− standard deviation to display their direction and magnitude consistently; their distributions were checked before parametric testing. Given the exploratory nature and the number of outcomes, no formal multiplicity correction was applied; findings were interpreted cautiously, and emphasis was placed on effect estimates and confidence intervals where available. MANOVA was selected because IL-6, ferritin, and CRP represent related components of the systemic inflammatory profile and were expected to be correlated. Their simultaneous analysis provided an omnibus assessment of whether the set of inflammatory outcomes varied with the explanatory variables, while reducing the inflation of type I error that would arise from fitting separate univariate models for each marker. Linearity, residual distributions, homoscedasticity, influential observations, and multicollinearity were assessed for model adequacy; variance inflation factors were used to evaluate multicollinearity. Pillai’s trace was selected as the multivariate test statistic because it is comparatively robust to unequal covariance matrices and departures from multivariate normality and is reported as the multivariate effect estimate. Two multivariable linear regression models examined factors associated with ferritin change, with unstandardized beta coefficients and 95% confidence intervals reported. Model A included age, sex, clinical group, baseline ferritin, baseline muscle mass, percentage muscle-mass change, MEDAS change, FLI change, CRP change, and the MCS score. Model B included age, sex, clinical group, total MEDAS score, and individual MEDAS items. Adjusted R^2^ was used to describe model explanatory performance. Analyses were complete-case/per-protocol with respect to attendance and data availability: participants without a final visit or required final laboratory data were excluded. Participants who attended the final visit remained in the analysis irrespective of imperfect adherence; consequently, this was not a strict adherent-only per-protocol analysis and not a true intention-to-treat analysis. No imputation was performed. Medication use, iron supplementation, reproductive or menopausal status, and other unmeasured causes of ferritin variation were not included as covariates. All tests were two-sided with alpha = 0.05. Analyses were conducted using STATA version 12.1 (StataCorp, College Station, TX, USA).

## 3. Results

### 3.1. Baseline Sample and Participant Characteristics

Baseline characteristics for the 251 participants included in cross-sectional comparisons (61 normal-weight controls and 190 participants with overweight/obesity) are shown in Table 1. Compared with normal-weight controls, the overweight/obesity sample had higher adiposity and less favorable metabolic and inflammatory characteristics. Omnibus comparisons across the three overweight/obesity clinical groups indicated heterogeneity in visceral fat and selected biochemical and health-related measures. Because adjusted post hoc comparisons were not available, the text does not attribute omnibus differences to specific pairs of groups.

### 3.2. Longitudinal Anthropometric, Metabolic, and Lifestyle Changes

Changes from baseline to the end of follow-up are summarized in Table 2. The omnibus between-group comparison was significant for MEDAS change (*p* = 0.045); no pairwise interpretation was made because adjusted post hoc comparisons were unavailable. Other anthropometric, biochemical, metabolic, and questionnaire changes did not show statistically significant between-group differences. Table 2 reports final-minus-baseline change scores because the longitudinal objective concerned within-participant response; the corresponding baseline distributions are presented in Table 1.

### 3.3. Inflammatory Markers at Baseline and During Follow-Up

Table 3 summarizes baseline inflammatory markers and longitudinal changes. Weight-status comparisons showed differences in CRP (*p* < 0.001), IL-6 (*p* = 0.003), fibrinogen (*p* = 0.003), and D-dimer (*p* = 0.037). Baseline ferritin differed significantly across the three overweight/obesity clinical groups (Kruskal–Wallis H = 15.21, *p* < 0.001, ε^2^ = 0.073). Baseline ferritin medians were 100.5 (53.5–178.5), 76.0 (34.0–137.0), and 149.5 (102.0–245.0) ng/mL in the healthy overweight, long-COVID, and MS groups, respectively. Post hoc comparisons showed a significant difference between the long-COVID and MS groups (Holm-adjusted *p* < 0.001), whereas the remaining pairwise comparisons were not statistically significant after adjustment.

Ferritin change also differed significantly across the three clinical groups (Kruskal–Wallis H = 6.52, *p* = 0.039, ε^2^ = 0.027), with mean changes of 8.0 ± 36.4, 3.3 ± 32.6, and −22.0 ± 58.8 ng/mL in the healthy overweight, long-COVID, and MS groups, respectively. Post hoc comparisons indicated significant differences between the MS group and both the healthy-overweight group (Holm-adjusted *p* = 0.0497) and the long-COVID group (Holm-adjusted *p* = 0.041), whereas the healthy-overweight and long-COVID groups did not differ significantly (Holm-adjusted *p* = 0.956). Changes in CRP, IL-6, fibrinogen, and D-dimer did not differ significantly across groups. Ferritin distribution according to clinical group and baseline and intervention are shown in Supplementary Figure S1.

### 3.4. Multivariable Analyses

The MANOVA showed a significant overall multivariate model (Pillai’s trace = 0.401, *p* < 0.001; Table 4). Sex (Pillai’s trace = 0.121, *p* < 0.001) and baseline muscle mass percentage (Pillai’s trace = 0.105, *p* < 0.001) were associated with the joint inflammatory profile. Clinical group, age, physical activity, MEDAS score, and muscle-mass change were not significant in the multivariate model.

Model A was statistically significant but had limited explanatory performance (adjusted R^2^ = 0.16). Ferritin change was associated with clinical group, baseline ferritin, MCS, muscle-mass change, MEDAS change, and FLI change (Table 5), whereas age, sex, and CRP change were not significant in Model A. Model B had an adjusted R^2^ of 0.25; age, sex, and MEDAS item 5 were associated with ferritin change, while the overall MEDAS score and other individual items were not. These coefficients describe associations and should not be interpreted as causal or predictive effects. Regarding age and sex, age was not associated with the joint inflammatory profile in MANOVA (*p* = 0.109), whereas sex was (*p* < 0.001). Neither variable was associated with ferritin change in Model A; both were associated in the exploratory item-level Model B. Age was retained as a continuous variable, and no post hoc age categories were created.

### 3.5. Ferritin According to Meat-Consumption Category

**Figure 2:**
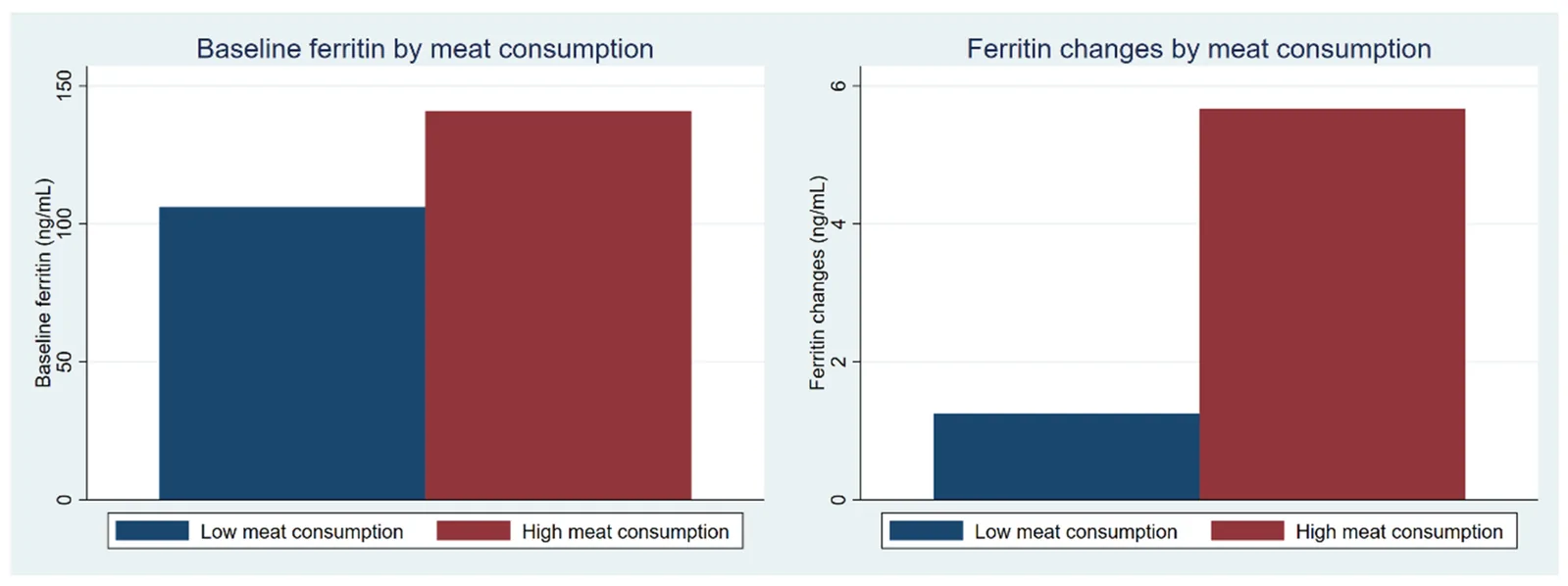
Distribution of baseline ferritin and ferritin changes according to baseline meat-consumption category. Individual-point dispersion is shown; inferential comparisons are reported in Supplementary Table S1.

Baseline ferritin concentrations were higher among participants classified as having higher meat consumption than among those with lower consumption (140.9 ± 114.0 vs. 106.1 ± 89.6 ng/mL; *p* = 0.026). By contrast, ferritin changes during follow-up did not differ significantly between the higher- and lower-consumption categories (5.7 ± 31.3 vs. 1.3 ± 56.4 ng/mL; *p* = 0.555), as illustrated in Figure 2 and detailed in Supplementary Table S1. Meat consumption was derived from the item MEDAS 5.

## 4. Discussion

This exploratory study compared inflammatory-metabolic phenotypes across healthy overweight, long-COVID overweight, and MS groups and examined longitudinal changes during an energy-restricted Mediterranean-style intervention. The principal observations were that baseline ferritin varied across clinical groups, ferritin change showed an omnibus between-group difference, and sex and muscle mass were associated with the joint inflammatory profile. Regression models also linked ferritin change with baseline ferritin, body-composition change, dietary adherence change, and liver-related status, but their modest adjusted R^2^ values indicate that most ferritin variability remained unexplained. Accordingly, the findings identify associations and hypothesis-generating patterns rather than causal or clinically predictive relationships. The prospective cohort design was selected to characterize naturally occurring heterogeneity across clinically defined groups receiving the same nutritional follow-up, rather than to estimate a causal treatment effect.

The less favorable profile observed in participants with overweight/obesity is consistent with previous descriptions of adipose-tissue dysfunction, immune-cell infiltration, and increased cytokine production [41,42]. Visceral adiposity has also been associated with hepatic insulin resistance, dyslipidemia, and acute-phase signaling [43,44,45]. Nevertheless, the present omnibus comparisons do not establish which overweight/obesity groups differed pairwise, and cross-sectional baseline differences may reflect age, sex distribution, treatment, or other clinical factors in addition to disease category.

Long COVID provides a distinct comparison because persistent symptoms may coexist with immune dysregulation, endothelial activation, altered coagulation, autonomic dysfunction, and metabolic abnormalities after SARS-CoV-2 infection. These pathways could affect CRP, IL-6, D-dimer, ferritin, fatigue, and physical activity, but their expression is heterogeneous and may diminish over time. In the present sample, long COVID did not produce a uniformly more adverse inflammatory profile than the other overweight groups. This finding argues against treating long COVID as a single high-inflammation phenotype and supports integrated interpretation with adiposity, body composition, hepatic status, and symptom burden. Post-acute sequelae following previous SARS-CoV-2 infection may involve persistent alterations in cytokine signaling, endothelial activation, coagulation, and hepatic-metabolic function. However, all long-COVID participants were assessed after resolution of the acute infection; therefore, the observed profiles should not be interpreted as biochemical or immunological effects of active SARS-CoV-2 infection. The lack of information on acute-treatment exposure, infection severity, and the exact time elapsed since infection nevertheless prevented attribution of these profiles specifically to the previous viral episode.

MEDAS scores changed during follow-up, with an omnibus difference across clinical groups, but the absence of adjusted pairwise tests prevents attribution to a specific group. Mediterranean dietary patterns have been associated with differences in inflammatory and metabolic markers in previous studies [46,47,48]. Energy restriction and adiposity changes may covary with these markers. Because the normal-weight control group was assessed only at baseline and adherence was imperfect, temporal changes cannot be separated confidently from concurrent care, natural disease evolution, other time-related factors, or the intervention itself.

Ferritin requires particularly cautious interpretation. In addition to reflecting iron stores, ferritin is an acute-phase reactant associated with obesity-related inflammation, insulin resistance, MS, and fatty liver [17,41,49,50,51,52]. The metabolic hyperferritinemia consensus distinguishes this condition from genetic iron overload and other causes and recommends evaluation beyond a single ferritin measurement [17]. The present study could not establish metabolic hyperferritinemia because transferrin saturation, hepcidin, serum iron and total iron-binding capacity, and direct liver-iron assessment were unavailable. Ferritin is also associated with sex, age, menstrual or menopausal status, heme-iron intake, supplementation, medication, alcohol use, liver injury, infection, and other clinical conditions. Thus, ferritin differences cannot be assigned specifically to inflammation or iron status, and longitudinal changes do not demonstrate an anti-inflammatory or iron-mediated dietary effect. Experimental work has proposed extracellular RNA/TLR3-dependent and independent signaling as a possible link between iron-related hepatocyte injury and inflammation [53], but no cellular, molecular, chelation, knockdown, or tissue-specific experiments were performed here; this pathway is only a contextual hypothesis and was not tested in this cohort. The higher baseline ferritin observed in the questionnaire-defined higher-meat category is compatible with a possible contribution of heme-iron exposure, but the cross-sectional comparison, binary MEDAS item, and absence of quantitative intake data prevent dose–response or causal interpretation.

The negative mean ferritin change observed in the MS group was part of the significant omnibus comparison and was significantly different from the changes observed in both the healthy-overweight and long-COVID groups in Holm-adjusted post hoc comparisons. The larger negative change in the MS group may partly reflect its higher baseline ferritin concentrations, although other factors, including hepatic-metabolic variation or unmeasured treatment and physiological factors, may also have contributed. The inverse coefficient for baseline ferritin in Model A is consistent with greater downward change among participants with higher starting concentrations, but it does not demonstrate that baseline ferritin predicts benefit from the intervention.

The multivariate associations of sex and muscle mass with the joint inflammatory profile are biologically plausible. Sex hormones, iron loss, fat distribution, and immune signaling can generate sex-related differences in both ferritin and inflammatory markers [54,55]. Skeletal muscle also acts as a metabolic and endocrine tissue, and lower muscle mass has been associated with systemic inflammation [56]. However, menopausal status and other reproductive variables were unavailable, limiting interpretation of the sex association. Age was not significant in the multivariate inflammatory model or ferritin-change Model A, although it was associated with ferritin change in the exploratory item-level Model B. Because age was analyzed continuously and subgroup sizes were limited, age categories were not introduced post hoc.

The regression findings suggest that ferritin change covaries with several metabolic and behavioral measures, but the adjusted R2 values of 0.16 and 0.25 indicate limited explanatory power and substantial residual variability. Model coefficients may also be unstable because of the number of candidate variables relative to group sizes and the absence of external validation. These models should therefore be regarded as exploratory and should not be used for individual prediction or treatment selection. The contrasting age and sex estimates across Models A and B further support cautious interpretation rather than claims of stable subgroup effects.

### 4.1. Strengths and Contribution

This study has strengths, including a clinically heterogeneous overweight/obesity sample and integrated assessment of body composition, biochemical markers, liver-related indices, and lifestyle factors. A potential contribution of this study is the simultaneous evaluation of ferritin, body composition, diet, hepatic indices, and several clinical inflammatory contexts, including long COVID. The findings help define questions for future controlled studies: whether baseline ferritin adds information beyond established metabolic and hepatic markers, whether ferritin responses differ reproducibly across clinical phenotypes, and whether changes persist after adjustment for iron status, medication, sex-specific physiology, and adherence. At present, baseline ferritin and body composition may support broader clinical characterization, but the evidence is insufficient to recommend them as standalone tools for selecting or monitoring a nutritional intervention.

### 4.2. Limitations

Several limitations are important. First, although a normal-weight control group was included for baseline comparisons, it did not undergo longitudinal follow-up; consequently, no contemporaneous longitudinal control group was available for the intervention period, precluding causal attribution of changes to the dietary program. Second, analyses were based on final-visit complete cases; participants lost to follow-up and those lacking final laboratory data were excluded, creating potential attrition bias. Adherence varied and was monitored by follow-up contacts and MEDAS rather than an objective biomarker. Third, detailed medication histories, including treatments received during acute COVID-19 and concomitant therapies for comorbidities, were not available for analysis. Participants with long COVID were evaluated after the acute infection had resolved, so acute-phase treatments were not contemporaneous with the nutritional intervention; however, residual or indirect treatment-related effects cannot be completely excluded. Infection severity and the exact time elapsed since SARS-CoV-2 infection were also not incorporated. Iron supplementation, menstrual or menopausal status, alcohol exposure, and other clinical causes of ferritin variation were not included, and assessors were not blinded to clinical group. Ferritin was measured by a single automated immunoassay without orthogonal confirmation, complete iron indices, hepcidin, or liver-iron imaging. Fourth, no functional experiments assessed ferritin or iron signaling, so no mechanism can be inferred. Fifth, although Holm-adjusted post hoc comparisons were performed for the principal ferritin analyses, no global multiplicity correction was applied across the broader set of exploratory analyses, increasing the possibility of chance findings. Sixth, bioelectrical impedance provides less precise body-composition assessment than reference imaging methods. Finally, the regression models explained only a modest proportion of ferritin variability and were not externally validated. These constraints require cautious, descriptive, association-based interpretation. Group assignment reflected the principal clinical phenotype and did not establish the absence of every coexisting condition.

## 5. Conclusions

In conclusion, baseline ferritin and body composition were correlates of heterogeneous inflammatory-metabolic profiles and ferritin changes during follow-up in adults with overweight/obesity and different clinical conditions. The absence of a longitudinal control arm, incomplete iron-status characterization, and absence of functional experiments preclude causal or mechanistic inference. Larger controlled studies with prespecified outcomes, comprehensive iron indices, assessment of confounding factors, objective adherence measures, and external validation are required before these markers can inform personalized nutritional decisions.

## Figures and Tables

**Table 1:** Baseline demographic characteristics, body composition, biochemical markers, metabolic indices, and lifestyle variables across study groups.

	Normal Weight	Healthy Overweight	Long-COVID Overweight	MS Overweight		
*n*	61	49	108	33	*p*-Value *	*p*-Value ^δ^
Sex, *n* (%)					<0.001	0.202
Men	12 (19.7)	15 (30.6)	19 (17.6)	19 (57.6)		
Women	49 (80.3)	34 (69.4)	89 (82.4)	14 (42.4)		
Men	18.46 (12)	23.08 (15)	29.23 (19)	29.23 (19)		
Women	26.34 (49)	18.28 (34)	47.85 (89)	7.53 (14)		
Age (years)	51.1 ± 9.8	56.9 ± 11.2	51.7 ± 7.5	62.9 ± 7.5	<0.001	0.007
Body composition						
Weight (kg)	61.5 (55.1;66.5)	84.1 (75.5;97.5)	82.6 (73.5;93.1)	87.6 (76.8;97.3)	0.258	<0.001
BMI (kg/m^2^)	22.9 ± 2.2	31.3 ± 3.5	31.3 ± 4.4	32.3 ± 4.0	0.444	<0.001
Fat mass (%)	27.1 (20.7;31.7)	37.6 (33.9;43.0)	39.3 (33.2;44.6)	37.6 (31.6;40.8)	0.464	<0.001
Visceral fat (%)	6.0 (4.0;7.0)	11.5 (9.5;15.0)	10.0 (8.0;12.5)	15.0 (11.0;19.0)	<0.001	<0.001
Muscle mass (kg)	40.6 (38.0;44.2)	47.3 (43.6;58.9)	47.2 (43.0;54.2)	52.1 (43.7;62.5)	0.143	<0.001
Waist (cm)	84.0 ± 8.2	107.7 ± 10.7	105.8 ± 12.2	110.5 ± 10.0	0.106	<0.001
Hip (cm)	95.9 ± 6.4	113.1 ± 9.3	112.5 ± 10.3	109.6 ± 7.9	0.230	<0.001
Blood biochemical markers						
Glucose (mg/dL)	89.0 (86.0;94.5)	94.0 (87.0;102.0)	95.0 (89.0;100.0)	102.0 (96.5;114.5)	<0.001	<0.001
Cholesterol (mg/dL)	199.6 ± 37.8	197.6 ± 25.1	201.6 ± 31.1	163.6 ± 35.2	<0.001	0.284
LDL-c (mg/dL)	117.6 ± 33.6	118.4 ± 20.8	120.6 ± 31.1	132.0 ± 32.7	<0.001	0.607
HDL-c (mg/dL)	64.0 (57.0;78.0)	59.0 (46.0;64.0)	55.0 (49.0;62.0)	47.0 (38.5;52.5)	<0.001	<0.001
Triglycerides (mg/dL)	71.0 (57.5;91.0)	100.0 (81.0;137.0)	101 (78;139)	107.5 (93.5;174.0)	0.133	<0.001
HbA1c (%)	5.3 (5.1;5.5)	5.4 (5.2;5.6)	5.3 (5.1;5.6)	5.8 (5.5;6.2)	<0.001	0.009
Insulin (U/mL)	6.0 (3.3;7.3)	9.8 (6.2,11.4)	10.0 (7.0;15.5)	9.4 (7.0;15.2)	0.283	<0.001
AST (U/L)	21.0 (17.0;25.5)	22.0 (19.0;24.0)	22.0 (18.0;28.0)	22.0 (19.0;27.5)	0.667	0.358
ALT (U/L)	19.0 (14.0;28.0)	22.0 (17.0;29.0)	24.0 (18.0;34.0)	28.0 (19.5;37.5)	0.149	0.002
Transferrin (mg/dL)	244 (218.5;266)	244 (223;267)	259 (237;278)	257 (235;279)	0.109	0.029
Hemoglobin (g/dL)	14.3 ± 1.1	14.9 ± 1.2	14.4 ± 1.1	15.2 ± 1.4	0.003	0.022
Metabolic indices						
TyG index (mg/dL)	8.1 (7.8;8.3)	8.5 (8.1;8.9)	8.4 (8.2;8.8)	8.6 (8.5;9.2)	0.013	<0.001
HOMA-IR	1.3 (0.7;1.7)	2.1 (1.4;2.8)	2.3 (1.6;3.5)	2.6 (1.7;3.6)	0.135	<0.001
FLI	14.7 (6.1;26.0)	66.7 (55.3;86.0)	65.5 (55.3;86.0)	77.4 (64.0;90.9)	0.121	<0.001
Lifestyle and health-related questionnaires						
MEDAS (points)	8.3 ± 1.8	7.9 ± 1.5	8.1 ± 1.9	8.6 ± 2.2	0.183	0.705
FACIT (points)	24.0 (17.0;36.0)	45.0 (33.0;47.0)	21.0 (15.5;29.5)	43.0 (32.0;48.0)	<0.001	0.088
SRQ20 (points)	8.0 (6.0;13.0)	3.0 (1.0;7.0)	10.0 (7.0;14.0)	3.0 (0.0;7.0)	<0.001	0.173
PCS12 (points)	36.0 (25.9;44.2)	49.3 (38.6;54.7)	30.2 (25.1;39.5)	42.4 (34.4;51.0)	<0.001	0.461
MCS12 (points)	42.8 (31.3;50.3)	48.9 (34.2;54.9)	34.7 (26.3;49.1)	50.1 (45.7;54.1)	<0.001	0.352
iPAQ (METs-min/wk)	982.5 (480;1588)	990 (594;1638)	604.5 (198;1188)	828 (396;1548)	0.039	0.161

* *p*-value among the three overweight/obesity groups; ^δ^ *p*-value between the normal-weight and combined overweight/obesity groups. For continuous variables, *p*-values were calculated using one-way ANOVA or the Kruskal–Wallis test for three-group comparisons and the independent-samples *t*-test or Mann–Whitney U test for two-group comparisons, according to distributional assumptions; categorical variables were assessed using the chi-squared test. MS, metabolic syndrome; BMI, body mass index; LDL-c, low-density lipoprotein cholesterol; HDL-c, high-density lipoprotein cholesterol; HbA1c, hemoglobin A1C; AST, aspartate aminotransferase; ALT, alanine aminotransferase; TyG, Triglyceride-Glucose index; HOMA-IR, Homeostatic Model Assessment-Insulin Resistance; FLI, Fatty Liver Index; MEDAS, Mediterranean Diet Adherence Screener; FACIT, Functional Assessment of Chronic Illness Therapy; SRQ20, Self-Report Questionnaire; PCS12, Physical Component Score; MCS12, Mental Component Score, iPAQ, International Physical Activity Questionnaire; METs, Metabolic Equivalent of Task.

**Table 2:** Final-minus-baseline changes in body composition, biochemical markers, metabolic indices, and lifestyle variables after the intervention according to overweight condition.

	Healthy Overweight	Long-COVID Overweight	MS Overweight	
*n*	49	108	33	*p*-Value
Body composition				
ΔWeight (kg)	−3.0 ± 4.1	−3.3 ± 5.0	−4.1 ± 4.9	0.559
ΔBMI (kg/m^2^)	−1.01 ± 1.5	−1.2 ± 1.8	−1.5 ± 1.7	0.569
ΔTotal fat (%)	−1.8 ± 2.4	−1.7 ± 3.2	−2.4 ± 3.2	0.551
ΔVisceral fat (%)	−0.8 ± 1.0	−0.7 ± 1.3	−0.9 ± 2.3	0.589
ΔMuscle mass (kg)	−0.7 ± 1.7	−0.6 ± 1.8	−0.6 ± 2.0	0.740
ΔWaist (cm)	−5.3 ± 4.5	−4.9 ± 5.6	−6.0 ± 4.8	0.580
ΔHip (cm)	−3.6 ± 3.8	−3.1 ± 4.4	−3.4 ± 4.1	0.810
Blood biochemical and hepatic markers				
ΔGlucose (mg/dL)	−4.1 ± 8.9	−3.1 ± 10.8	0.5 ± 22.6	0.468
ΔTotal cholesterol (mg/dL)	−3.0 ± 26.3	−1.5 ± 32.6	1.4 ± 25.2	0.520
ΔLDL-c (mg/dL)	−0.3 ± 20.5	0.2 ± 30.4	−1.7 ± 25.8	0.960
ΔTriglycerides (mg/dL)	−11.5 ± 29.3	−4.5 ± 45.6	2.7 ± 39.3	0.321
ΔhbA1c (%)	−0.9 ± 0.2	−0.1 ± 0.2	−0.2 ± 0.6	0.604
ΔInsulin (U/mL)	0.2 ± 4.6	−3.1 ± 9.6	1.2 ± 9.0	0.141
ΔAST (U/L)	0.8 ± 4.0	0.2 ± 13.1	−0.7 ± 4.2	0.172
ΔALT (U/L)	−1.6 ± 8.6	−1.6 ± 16.4	−3.4 ± 6.2	0.646
ΔTransferrin (mg/dL)	−3.4 ± 16.7	−4.4 ± 25.8	−3.4 ± 19.2	0.825
ΔHemoglobin (g/dL)	−0.1 ± 0.6	−0.1 ± 1.3	0.01 ± 0.8	0.933
Metabolic indices				
ΔTyG index	−0.14 ± 0.3	−0.07 ± 0.4	0.01 ± 0.4	0.341
ΔHOMA-IR	0.01 ± 1.3	−1.03 ± 3.9	0.78 ± 4.6	0.134
ΔFLI	−9.7 ± 11.5	−8.3 ± 15.5	−11.8 ± 14.3	0.518
Lifestyle and health-related questionnaires				
ΔMEDAS	1.5 ± 1.7	1.2 ± 2.0	0.8 ± 1.5	0.045
ΔFACIT	1.3 ± 9.7	3.2 ± 9.8	1.1 ± 7.9	0.552
ΔSRQ20	−1.4 ± 3.0	−1.01 ± 3.8	−1.0 ± 3.0	0.726
ΔPCS12	0.6 ± 10.2	3.2 ± 10.0	4.7 ± 9.9	0.178
ΔMCS12	3.0 ± 11.5	2.1 ± 12.4	−1.5 ± 10.3	0.499
ΔiPAQ (METs-min/wk)	−717.7 ± 1054.1	−229.5 ± 1926.2	−657.2 ± 2392.1	0.073

MS, metabolic syndrome; BMI, body mass index; LDL-c, low-density lipoprotein cholesterol; HbA1c, hemoglobin A1C; AST, aspartate aminotransferase; ALT, alanine aminotransferase; TyG, Triglyceride-Glucose index; HOMA-IR, Homeostatic Model Assessment-Insulin Resistance; FLI, Fatty Liver Index; MEDAS, Mediterranean Diet Adherence Screener; FACIT, Functional Assessment of Chronic Illness Therapy; SRQ20, Self-Report Questionnaire; PCS12, Physical Component Score; MCS12, Mental Component Score, iPAQ, International Physical Activity Questionnaire; METs, Metabolic Equivalent of Task. Values in Table 2 are final-minus-baseline changes and are presented as mean +/− SD. Between-group *p*-values were obtained using one-way ANOVA after assessment of change-score distributions.

**Table 3:** Baseline values and change in inflammatory markers in METAINFLAMMATION-CM cohort.

	Normal Weight	Healthy Overweight	Long-COVID Overweight	MS Overweight		
*n*	61	49	108	33	*p*-Value *	*p*-Value ^δ^
Baseline inflammatory markers						
CRP (mg/L)	0.9 (0.9;1.0)	2.0 (0.9;5.0)	1.8 (0.9;4.1)	3.0 (0.9;6.0)	0.297	<0.001
IL-6 (pg/mL)	2.6 (2.6;2.8)	2.6 (2.6;3.8)	2.6 (2.6;3.4)	2.6 (2.6;3.9)	0.796	0.052
Ferritin (ng/mL)	72.5 (38.5;127.5)	100.5 (53.5;178.5)	76.0 (34.0;137.0)	149.5 (102;245)	<0.001	0.100
Fibrinogen (mg/dL)	324 (276;364)	365.0 (310;425)	342.5 (306;385)	335 (305;417)	0.364	0.003
D-Dimer (ng/mL)	246 (165;370)	346 (220;469)	291.5 (202;432)	294 (250;425)	0.460	0.037
Changes in inflammatory markers						
ΔCRP (mg/L)		−1.8 ± 9.9	0.2 ± 4.1	−1.0 ± 4.4	0.326	NA
ΔIL-6 (pg/mL)		−0.3 ± 1.8	0.3 ± 1.9	−0.4 ± 2.1	0.652	NA
ΔFerritin (ng/mL)		8.0 ± 36.4	3.3 ± 32.6	−22.0 ± 58.8	0.039	NA
ΔFibrinogen (mg/dL)		−14.8 ± 53.1	−11.0 ± 57.2	−19.8 ± 74.5	0.992	NA
ΔD-Dimer (ng/mL)		98.9 ± 708.8	47.4 ± 374.5	26.6 ± 157.5	0.855	NA

* *p*-value among the three overweight/obese groups. ^δ^ *p*-value between the normal-weight and overweight groups. MS, metabolic syndrome; CRP, C-reactive protein; IL-6, interleukin-6. NA, not applicable.

**Table 4:** Multivariate analysis of factors associated with inflammatory markers.

Effect	Test Statistic	Value	F	df	*p*-Value
Model	P	0.401	24.0	489.0;3.1	<0.001
ΔMuscle mass (%)	P	0.012	3.0	161.0;0.7	0.574
Muscle mass (%)	P	0.105	3.0	161.0;6.3	<0.001
Group	P	0.031	6.0	324.0;0.9	0.535
Sex	P	0.121	3.0	161.0;7.4	<0.001
Age (years)	P	0.037	3.0	161.0;2.1	0.109
METs (min/wk)	P	0.017	3.0	161.0;1.0	0.418
MEDAS14	P	0.018	3.0	161.0;1.0	0.396

P, Pillai’s trace; METs, Metabolic Equivalent of Task; MEDAS14, validated Mediterranean Diet Adherence Screener with 14 questions.

**Table 5:** Linear regression model of factors associated with changes in ferritin levels.

	Ferritin Changes		
	β Coefficient (95% CI)	*p*-Value	Adjusted R^2^
Model A		<0.005	0.16
Age (years)	0.03 (−0.79;0.79)	0.947	
Sex	7.06 (−10.14;24.25)	0.419	
Type of disease	−5.55 (−11.08;−0.03)	0.049	
Baseline ferritin (ng/mL)	−0.12 (−0.20;−0.04)	0.003	
MCS (points)	−0.72 (−1.28;−0.16)	0.012	
Muscle mass change (%)	3.91 (0.94;6.88)	0.010	
MEDAS change (points)	4.16 (0.28;8.04)	0.036	
FLI change	0.87 (0.25;1.48)	0.006	
CRP change (mg/L)	0.95 (−0.12;2.03)	0.082	
Model B		<0.005	0.25
Age (years)	2.18 (0.64;3.71)	0.006	
Sex	−90.36 (−120.95;−59.77)	<0.001	
Type of disease	−7.45 (−18.25;3.36)	0.175	
MEDAS (points)	2.67 (−16.70;22.05)	0.786	
MEDAS 1 (points)	98.51 (−35.90;232.92)	0.150	
MEDAS 2 (points)	−18.67 (−47.89;10.54)	0.209	
MEDAS 3 (points)	8.37 (−24.22;40.96)	0.613	
MEDAS 4 (points)	−18.38 (−56.12;19.37)	0.338	
MEDAS 5 (points)	−41.63 (−76.88;−6.37)	0.021	
MEDAS 6 (points)	−20.27 (−69.41;28.87)	0.417	
MEDAS 7 (points)	1.70 (−38.30;41.69)	0.933	
MEDAS 8 (points)	20.40 (−72.03;112.83)	0.664	
MEDAS 9 (points)	−34.95 (−79.33;9.42)	0.122	
MEDAS 10 (points)	−12.22 (−51.67;27.24)	0.542	
MEDAS 11 (points)	−8.00 (−35.62;19.62)	0.568	
MEDAS 12 (points)	−10.60 (−40.82;19.62)	0.489	
MEDAS 13 (points)	15.39 (−26.65;57.42)	0.471	
MEDAS 14 (points)	14.62 (−18.10;47.34)	0.379	

CI, confidence interval; MCS, Mental Component Summary; MEDAS, Mediterranean Diet Adherence Screener; FLI, Fatty Liver Index; CRP, C-reactive protein.

## Data Availability

The clinical, biochemical, and follow-up data supporting the findings of this study are recorded and securely maintained within SELENE-PdH, the institutional Electronic Health Record (EHR) system of Hospital Universitario Puerta de Hierro Majadahonda. The study was conducted within the METAINFLAMMATION-CM project (Y2020/BIO-6600) and was approved by the Research Ethics Committee of Hospital Universitario Puerta de Hierro Majadahonda (PI 164/21). The derived data generated from the study participants are publicly available through the NCBI Sequence Read Archive and Metadata (SRA) under BioProject accession number PRJNA1279632. Additional information and, where applicable, supporting data may be made available by the corresponding author upon reasonable request, subject to institutional and ethical approval and applicable data protection regulations. The project’s protocol and procedures fulfilled all the regulations and requirements of the Spanish Agency for Research.

## Supplementary Materials

The following supporting information can be downloaded at: https://www.mdpi.com/article/10.3390/nu18183044/s1, Table S1. Baseline ferritin levels and changes in ferritin according to meat consumption; Figure S1. Ferritin distribution according to clinical group. (A) Baseline ferritin concentrations and ferritin changes during follow-up across the healthy-overweight, long-COVID overweight, and metabolic-syndrome overweight groups. Boxplots show the median and interquartile range, with individual participant values overlaid. (B) Distribution of baseline ferritin and ferritin changes according to baseline meat-consumption category.

## Abbreviations

The following abbreviations are used in this manuscript:

ALT	alanine aminotransferase
AST	aspartate aminotransferase
BMI	body mass index
CI	confidence interval
CRP	C-reactive protein
FACIT	Functional Assessment of Chronic Illness Therapy
FLI	Fatty Liver Index
HbA1c	glycated hemoglobin
HDL-c	high-density lipoprotein cholesterol
HOMA-IR	Homeostatic Model Assessment of Insulin Resistance
HRQoL	health-related quality of life
IL-6	interleukin-6
IPAQ	International Physical Activity Questionnaire
LDL-c	low-density lipoprotein cholesterol
MCS	Mental Component Summary
MEDAS	Mediterranean Diet Adherence Screener
MET	Metabolic Equivalent of Task
MS	metabolic syndrome
PCS	Physical Component Summary
SD	standard deviation
SRQ-20	Self-Reporting Questionnaire-20
TG	Triglycerides
TNF-α	tumor necrosis factor-alpha
TyG	Triglyceride-Glucose index
VIF	variance inflation factor
WC	waist circumference
