# Supplementary material for: Baseline Ferritin and Body Composition as Correlates of Longitudinal Metabolic Changes During an Energy-Restricted Mediterranean-Style Intervention Across Three Overweight Phlogistic-Related Clinical Conditions
Source: Nutrients. 2026 Sep 17;18(18):3044. doi: 10.3390/nu18183044 (PMC13610525; doi:10.3390/nu18183044)

Supplemental Table S1. Baseline ferritin levels and changes in ferritin according to meat consumption.

	Low meat consumption	High meat consumption	
**n**	**108**	**78**	**p-value**
Baseline ferritin (ng/ml)	106.1±89.6	140.9±114.0	**0.026**
ΔFerritin (ng/ml)	1.3±56.4	5.7±31.3	0.555

Supplemental Figure S1. Ferritin distribution according to clinical group. **(A)** Baseline ferritin concentrations and ferritin changes during follow-up across the healthy-overweight, long-COVID overweight, and metabolic-syndrome overweight groups. Boxplots show the median and interquartile range, with individual participant values overlaid. **(B)** Distribution of baseline ferritin and ferritin changes according to baseline meat-consumption category.

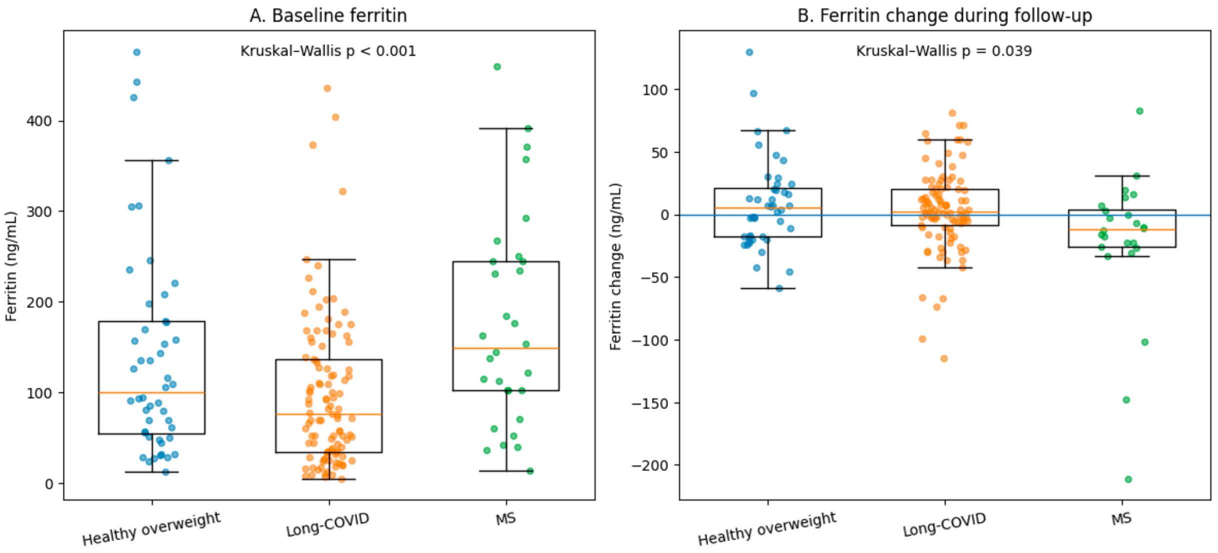

Supplement: Supplementary file 1 [file nutrients-18-03044-s001.zip › nutrients-4500685-supplementary.pdf]
